# Editorial: How do Emotions and Feelings Regulate Physical Activity?

**DOI:** 10.3389/fpsyg.2017.01145

**Published:** 2017-07-11

**Authors:** Darko Jekauc, Ralf Brand

**Affiliations:** ^1^Institute for Sport Science, Humboldt University of Berlin Berlin, Germany; ^2^Department for Sport and Exercise Psychology, University of Potsdam Potsdam, Germany

**Keywords:** physical activity, exercise, emotions, feelings, affect, regulation, implicit, enjoyment

Intuitively, it is obvious that emotions and feelings influence our behavior. Therefore, it can be assumed that activities we like will be pursued with greater engagement and persistence than activities we do not like. When people feel good during or after physical activity, it can be expected that they will be more prone to repeat that behavior.

Despite this apparent relationship, affective states have been long ignored as a possible determinant of physical activity. In the last 15 years, however, the interest of researchers in affective determinants of physical activity erratically increased. Two reviews and meta-analyses have been conducted to summarize the empirical evidence for this relationship showing that only few longitudinal studies with remarkable methodological shortcomings exist (Rhodes et al., 2009; Rhodes and Kates, 2015). Furthermore, these reviews reveal that the underlying mechanisms are not well understood. For example, in 19 out of 20 identified experimental studies in the meta-analysis by Rhodes et al. (2009) affective variables were not influenced by the current intervention strategies. Only few studies used interventions explicitly targeting emotions and affective states to increase exercise adherence (Jekauc, 2015). Overall, it can be concluded that some empirical endeavors exist to analyze the role of affective states as determinants of physical activity but there are no fruitful theoretical frameworks to inspire research. The aim of this Research Topic is to make a contribution to close this research gap. It yielded 16 articles embedded in five strands of research. Firstly, three articles examined the role of affective variables within the scope of the social-cognitive perspective. Secondly, two articles proposed new theoretical approaches to understand the affective mechanisms in promotion of physical activity. Thirdly, four articles examined the determinants of affective variables. Fourthly, five articles examined the effects of affective variables on consecutive physical activity on a daily level using the methodology of ambulatory assessment. Fifthly, two articles examined non-conscious mechanisms by studying automatic evaluations. These five research strands are presented below.

One reason for the above mentioned shortage of theoretical development is that social-cognitive models dominated physical activity research for several decades (Jekauc et al., 2015). These models focused on cognitive mechanisms and neglected the role of affective states and emotions. More recently, researchers operating within the scope of *social-cognitive perspective* began to examine the role of affective variables. Klusmann et al. examined the role of emotional outcome expectancies within the framework of social-cognitive models and found that fulfillment of emotional outcome expectancies emerges as a significant predictor of adoption and maintenance of physical activity. Furthermore, two studies examined the role and the interdependencies of affective variables and self-efficacy in physical activity setting. Ungar et al. show that physical activity enjoyment was as strong predictor of physical activity level as self-efficacy in cancer patients. This study suggests that physical activity enjoyment has an independent and equal effect on physical activity level as self-efficacy. At the same time, Matsuo et al. provide evidence that post-exercise self-efficacy is related to subjective and objective measures of affectivestates indicating that self-efficacy and affective states are mutually dependent on each other. These three studies suggest that the effects of affective variables on physical activity are independent of self-efficacy whereas they both interact with each other at the same time. These studies imply that theoretical developments to extend social-cognitive models might be a promising way to examine the role of emotions and affects in physical activity maintenance.

Within the scope of this Research Topic, *alternative theoretical approaches* have been developed to understand the role of affective variables on physical activity. Lee et al. propose an evolutionary perspective hypothesizing that affective response to exercise will be more positive when the perceived immediate utility of the physical activity is high. The authors argue that promotion of activities which have an inherent utility like walking for transportation could be an effective strategy to increase physical activity levels. Furthermore, Murphy and Eaves discuss the hedonic perspective to explain the effects of affective variable on physical activity and point to the indiscriminant use of hedonistic terms in different areas of psychology and philosophy. Both perspectives provide innovative ideas which could be fertile for development of new intervention approaches beyond social-cognitive models.

A group of researchers examined with different methodological approaches the *determinants of affective variables*. Wienke and Jekauc examined the conditions of exercise under which enjoyment and positive emotions emerge. They discovered four factors, “perceived competence,” “social interaction,” “novelty experience,” and “perceived physical exertion” which are associated with positive emotions. Independently, Sudeck et al. examined how two of these factors “perception of competence” and “perceived exertion” as well as “affective response to exercise” influence the development of affective attitude. The authors found out that perceptions of competence have a direct effect on changes in affective attitude whereas the effect of perceived exertion was indirect via positive activation influences on the development of affective attitude. Furthermore, Pilcher and Baker showed that physical activity at work on a stationary bike with a low level of exertion led to increases in positive affective states compared with the traditional work situation of sitting at an office desk. This study illustrates that low intensity physical activity at an active workstation increases positive feelings during work without having a negative effect on cognitive tasks. Rhodes and Mistry examined the reasons for anticipated regret over missing regular physical activity. The authors identified three dominating themes “missed opportunity to obtain the benefits of physical activity,” “shame and guilt for not being able to follow-through with one's goals,” and “perceived pressure from others.” These studies can be used as a starting point for the development of innovative emotion-based interventions to promote physical activity.

An innovative methodological approach that examines the relationship between physical activity and affective states on a daily basis is the *ambulatory assessment method*. In a review conducted by Liao et al., six studies were identified that examined the relationship between affective states and subsequent physical activity. The findings suggest that positive affective states, for a few hours afterwards, were positively associated with physical activity while negative affective states had no significant association. These findings were confirmed in the studies by Niermann et al., Schöndube et al., Reichert et al., and Kanning and Schoebi which consistently showed that positive feelings and moods were positively associated with subsequent physical activity. In contrast to the findings of Liao et al., Niermann et al. found that negative affect decreased subsequent MVPA. This number of studies reflects the usefulness of the ambulatory assessment method for the examination of the relationship between affective states and physical activity. It illustrates how technological developments influence research in physical activity.

Another theoretical approach exceeding social-cognitive models represents the analysis of non-conscious processes such as *automatic evaluations*. Antoniewicz and Brand discovered that automatic evaluations of exercise at the beginning of an exercise program significantly predicts exercise adherence in the upcoming months. The study reveals the significance of non-conscious processes for maintenance of physical activity. Furthermore, Rebar et al. establish population-level evidence of the most common word stimuli for physical activity and pleasantness. These can be used as resources for response latency measurement tasks of automatic evaluations and as tools to enhance automatic evaluations of physical activity in evaluative conditioning tasks.

The aim of this Research Topic was to stimulate and pool systematic research on the role of emotions and feelings as predictors of physical activity. Several theoretical and empirical works contributed toward deepening the understanding of the working mechanisms.

## Author contributions

All authors listed have made a substantial, direct and intellectual contribution to the work, and approved it for publication.

### Conflict of interest statement

The authors declare that the research was conducted in the absence of any commercial or financial relationships that could be construed as a potential conflict of interest.
